# Multivariate variable selection in N-of-1 observational studies via additive Bayesian networks

**DOI:** 10.1371/journal.pone.0305225

**Published:** 2024-08-26

**Authors:** Christian Pascual, Keith Diaz, Sonia Jain

**Affiliations:** 1 Herbert Wertheim School of Public Health and Human Longevity Science, University of California, San Diego, San Diego, CA, United States of America; 2 Center for Behavioral Cardiovascular Health, Columbia University Medical Center, New York, NY, United States of America; TU Wien: Technische Universitat Wien, AUSTRIA

## Abstract

An N-of-1 observational design characterizes associations among several variables over time in a single individual. Traditional statistical models recommended for experimental N-of-1 trials may not adequately model these observational relationships. We propose an additive Bayesian network using a generalized linear mixed-effects model for the local mean as a novel method for modeling each of these relationships in a data-driven manner. We validate our approach via simulation studies and apply it to a 12-month observational N-of-1 study exploring the impact of stress on daily exercise engagement. We demonstrate the improved performance of the additive Bayesian network to recover the underlying network structure. From the empirical study, we found statistically discernible associations between reports of stress and physical activity on a population level, but these associations may differ at an individual level.

## Introduction

N-of-1 designs are studies of a single individual through repeated, prospective measurement over time. N-of-1 designs can be classified into two main types; experimental N-of-1 designs which incorporate an interventional component while observational N-of-1 designs do not. Observational N-of-1 designs are used to study multiple features of interest and the inter-relationships between these variables simultaneously [1]. Observational N-of-1 designs are relatively novel, in which the first studies were published in 2013 to assess the validity of behavioral theories [2, 3]. Currently, observational N-of-1 designs have been conducted primarily in the fields of health psychology and behavioral medicine [4–6]. This niche stems from innovations in mobile health and wearable technologies which have facilitated convenient, objective measurement of physical activity and other health behaviors [7].

Several exposure-outcome relationships are considered concurrently in observational N-of-1 designs which can require more flexible models compared to those used in experimental N-of-1 settings. Models used in experimental N-of-1 designs may be employed in observational settings, but the exposure-outcome relationships (causal and association) need to be established *a priori*, which requires drawing on existing research, theory or expert guidance. Examining these relationships is further complicated by the fact that they may vary at the individual level. In the absence of such evidence, this motivates a data-driven variable selection process to identify these relationships. Several variable selection methods exist, but these methods do not adequately address the longitudinal nature of observational N-of-1 data [8].

To address this gap, we propose applying additive Bayesian networks (ABNs) as an analytic method for modeling an N-of-1 observational study. ABNs are an extension of Bayesian networks that use additive models for the local mean of each variable. ABNs can be viewed as a multidimensional regression model, in which each variable in the node set of interest is potentially dependent on some subset of the whole. Since data aggregated from N-of-1 designs are clustered within an individual, we can account for this by utilizing mixed-effects models for the local distributions. Our approach allows us to model relationships on both an individual level and population level, which can then be used to design future interventions (i.e., series of N-of-1 trials) based on the learned network structure. Some of these relationships may subsequently be investigated as possible causes or triggers through an n-of-1 trial.

The paper is organized as follows. First, we formally describes Bayesian networks and discusses how they can be modified to form an ABN. Next, we describe the parameters and metrics used to compare candidate variable selection models in a simulation study. Then we apply our approach to data from a 12-month observational N-of-1 study exploring the individual average impact of stress on daily exercise engagement. The paper concludes with a discussion.

## Methodology

### Bayesian networks

Bayesian networks (BN) are a class of probabilistic graphical models that represent the dependencies present in a given set of random variables [9, 10]. A Bayesian network is a graph G=(X,E) that consists of a set of *N* nodes **X** = (*X*_1_, …, *X*_*N*_) and a set of directed edges *E*. Each node represents a random variable *X*_*i*_, and a directed edge represents a conditional dependence between two distinct variables, *X*_*i*_ and *X*_*j*_ (where *i* ≠ *j*). The direction of the edge defines a parent-child relationship between these two nodes. *X*_*j*_ is said to be a parent of *X*_*i*_ if there exists a directed edge in *E* that starts at *X*_*j*_ and points to *X*_*i*_. We denote the set of parents of a node *X*_*i*_ as *Pa*(*X*_*i*_).

Bayesian networks are directed, acyclic graphs (DAG), so cycles are not allowed in G. Bayesian networks have been used in a wide variety of real-world applications, including medical diagnosis, epidemiology, and manufacturing control [11–13]. Despite its name, Bayesian networks can facilitate both Bayesian and frequentist inference. In the present article, we use frequentist modeling. Many authors have previously used Bayesian networks to facilitate causal discovery in observational data, but we note that this is not the goal of this paper [14]. An interested reader can refer to [15] for more information on Bayesian networks.

The joint distribution of **X**, denoted as P(X), is typically difficult to estimate, but Bayesian networks simplify this task by expressing P(X) as a product of each node’s conditional probability on their parents. Eq 1 expresses this factorized representation:

$$\begin{array}{c} P\left(X_{1},X_{2}\ldots ,X_{n}\right)=\prod_{i=1}^{n}P\left(X_{i}|Pa\left(X_{i}\right)\right) \end{array}$$ (1)

That is, the global distribution of **X** can be expressed as the product of many local distributions due to the mapping between the edge set *E* and the dependence relationships of P(X) [9]. Depending on the form of the conditional probability in Eq 1, we may construct different types of Bayesian networks.

### Types of Bayesian networks

The classical BN types are the discrete Bayesian network (DBN) and the Gaussian Bayesian network (GBN), which are characterized by the fact that their node set **X** is entirely categorical or continuous, respectively. For the DBN, each $P(X_{i}|Pa\left(X_{i}\right))$ is modeled as a conditional probability table. In a GBN, the conditional distributions are modeled as Gaussian random variables, in which the mean is a linear combination of the parent nodes. The local mean of the GBN is shown in Eq 2, where *card*(*W*) represents the cardinality of some set *W*.

$$\begin{array}{c} X_{i}|Pa\left(X_{i}\right)=\beta_{i0}+\sum_{k=1}^{card(Pa\left(X_{i}\right))}\beta_{ik}Pa_{k}\left(X_{i}\right)+\epsilon ,\;\epsilon ∼N\left(0,\sigma_{i}^{2}\right) \end{array}$$ (2)

Note in Eq 2 that *i* indexes each node in **X**, and *k* indexes each node in the parent node set *Pa*(*X*_*i*_). From a practical perspective, a weakness of the DBN and GBN is that they can only be applied to a limited range of data sets. To encompass a wider set of possible data sets, we consider a different type of Bayesian network in this paper.

The additive Bayesian network (ABN) is a special case of a BN that uses an exponential family for the local mean in Eq 1 [16]. Eq 3 describes the local mean of the ABN.

$$\begin{array}{c} g(E[X_{i}|Pa\left(X_{i}\right)])=\beta_{i0}+\sum_{k=1}^{\left|Pa(X_{i})\right|}\beta_{ik}Pa_{k}\left(X_{i}\right) \end{array}$$ (3)

where *g* is an appropriate link function for the child node *X*_*i*_ (e.g., the logit function for a binary node). By taking advantage of the exponential family, ABNs enable the use of mixed data types for both child and parent nodes in modeling the local means. ABNs are *additive* in the sense that the mean associations of the parent nodes with the child node are linearly summed on the scale of the exponential family link function. Intuitively, an ABN can be viewed as a set of generalized linear models, subject to restrictions on the edge set *E* that preserve the network’s acyclicity. Although they are relatively new compared to DBNs and GBNs, ABNs have been applied in the fields of epidemiology, veterinary medicine and nutrition [17–20].

The flexibility of choice in the local mean makes ABNs a particularly useful modeling choice for observational N-of-1 data. ABNs can be modified to accommodate clustering in the data (e.g., per individual) by using an appropriate generalized mixed-effects model (GLMM), as is recommended in the seminal guide by Kravitz *et. al* [21]. Thus, ABNs can accommodate multiple exposure-outcome relationships simultaneously while allowing for modeling on both the individual and population level.

### Network learning

The process of constructing a BN is called network learning, and it occurs in two phases: a structure learning phase and a parameter learning phase. Structure learning is the process of learning *E*, the set of edges or parent-child relationships in the network. The time for an exhaustive search of all possible network structures grows exponentially in the number of nodes in the network, so approximation algorithms are often used instead for computational tractability [22]. These structure learning algorithms can be approximately divided into three approaches: constraint-based, score-based or a hybrid of the two. Constraint-based algorithms learn the BN structure via conditional independence tests, while score-based algorithms use general optimization techniques to learn the structure. For a given network, the scoring algorithm evaluates some metric such as the Bayesian Information Criterion (BIC) and changes the edge set to optimize this metric. Additionally, experts may also specify the existence or absence of a node based on their knowledge [23]. In particular, ABN structures are learned through a score-based algorithm. Once a structure has been chosen, the parameters of the network are learned by applying the relevant optimization algorithm to each local mean.

### Simulation study

N-of-1 data are collected by taking multiple observations (i.e., repeated measures) per individual. Hence, we propose that the ABN using a GLMM for the local mean is an ideal candidate model for analyzing observational N-of-1 data across individuals, clustered by individual. Hereafter, we refer to this model as the *mixed-effects ABN*. For all local distributions in the mixed-effects ABN, a random intercept will be used and each parent node is given a random slope. Eq 4 gives the local mean of the node *X*_*i*_ for the *l*^th^ individual in the mixed-effects ABN:

$$\begin{array}{c} g(E[X_{i}|Pa\left(X_{i}\right),b_{il}])=\left(\beta_{i0}+b_{i0l}\right)+\sum_{k=1}^{\left|Pa(X_{i})\right|}\left(\beta_{ik}+b_{ikl}\right)Pa_{k}\left(X_{i}\right) \end{array}$$ (4)

where **b**_*l*_ = {*b*_*i*0*l*_, *b*_*i*1*l*_, …, *b*_*ikl*_} is the vector of random effects of the *l*^th^ individual. This parameterization reflects a belief that each parent node will have a different association with the child node for each individual.

#### Competing structure learning methods

In our simulation study, we compared the mixed-effects ABN against three other candidate models which could reasonably be used to analyze this observational N-of-1 data. The first competing model was an ABN which used a GLM for the local distribution; this model will be referred to as the *naive ABN* since it does not account for the individual level clustering inherent in observational N-of-1 data measured across individuals (i.e., a “no pooling” approach versus the “partial pooling” of GLMMs). The second competing model was a set of GLMMs constructed using forward selection. This set was indexed by each node *X*_*i*_ in **X**, in which that node acts as the outcome for that GLMM. The third competing model is similar to the second, but instead used backward selection. We refer to these models as the *forward stepwise* and *backward stepwise* models, respectively. We used the BIC as the scoring criterion in the structure learning phase for the naive and mixed-effects ABNs. We also use the BIC as the criterion for learning the set of models for the stepwise methods. Once the parent-child relationships were learned, the parameters for all models were learned via standard optimization algorithms for mixed-effects models. Note that the forward and backward stepwise models are not Bayesian networks; they account for the clustering in the data, but are not subject to the acyclic requirement of the BNs. The bnlearn package was used to construct the naive and mixed-effects ABNs, and the buildmer package was used to construct the mixed-effects stepwise models [24, 25].

We varied the structure in the underlying network and the data generated from these networks based on different characteristics, as outlined in Table 1. These parameters captured a wide range of scenarios we might encounter in a real-world context. Given a combination of parameters, we generated five independent ABNs to capture possible variation in the underlying networks. Then, for each ABN, we generated a single dataset. 108 parameter configurations were considered, which resulted in a total of 540 data sets that were evaluated in our study. We varied the number of individuals contributing data, the size of the node set **X**, the number of observations generated per individual, and the average number of parents. Note that the average number of parents is a measure of network density. These parameters were validated in a simulation study by Scutari *et. al* for comparing models used for structure learning in BNs [26]. The underlying networks and data were generated using the abn R package [27].

**Table 1:** Parameter values used to construct data-generating structures in simulation study.

Simulation Parameter	Values
Number of individuals contributing to data	2, 5, 10, 20
Number of nodes in the network	10, 20, 30
Number of observations generated per individual	30, 45, 60
Average number of parents per child	1, 2, 4

For a given dataset and structure learning method, we learned a network structure and evaluated several performance metrics to assess their ability to reconstruct the underlying BN. BNs are DAGs, so we considered not only the presence or absence of an edge, but also its parent-child orientation. Furthermore, randomness in the network generation process and different parameter configurations can result in varying cardinality of the true edge set. Let $\hat{E}$ represent the learned edge set. The first metric we considered was the ratio of number of matched (i.e. correctly identified) edges to the cardinality of the true edge set. An edge *ϵ* is correctly identified if it is in both the true and estimated BN (i.e., $\epsilon \in E\cap \hat{E}$) *and* has the correct parent-child orientation. Normalizing by the cardinality of the true edge set, *card*(*E*), allows for comparability across parameter configurations. The second metric was the ratio of the Structural Hamming Distance (SHD) between the true and estimated BN to *card*(*E*). The SHD measures the number of changes needed to convert one network into another and is calculated as the sum of missing edges, extraneous edges, and flipped edges. Lower SHD values are better since they indicate two networks are “closer” in structure. A missing edge was an edge that was neither in the learned network nor the true network ($\epsilon \in E^{C}\cap {\hat{E}}^{C}$) where *C* denotes the complement. An extraneous edge was defined as an edge that appeared in the learned network, but not in the true network ($\epsilon \in E^{C}\cap \hat{E}$). Finally, a flipped edge was an edge that is in $\hat{E}$ ($X_{i}\to X_{j}\in \hat{E}$), but its orientation was incorrect (*X*_*j*_ → *X*_*i*_ ∈ *E*). In general, a flipped is preferred to a missing edge since it indicates a model can correctly identify a relationship in the network, albeit not its orientation. We expect the stepwise models to correctly identify edges in the true model, but will use the incorrect parent-child orientation due to the fact they are not subject to acyclicity. Our paper focuses on structure learning, so we did not consider parameter-based performance metrics such as bias or mean-squared error.

## Results

### Simulation results

#### Model performance

Table 2 shows the performance of the different structure learning methods, aggregated across the different permutations of underlying networks configurations. The mixed-effects ABN achieved the highest proportion of matched edges at 32.4%, followed by the forward stepwise model (28.2%) and backward stepwise model (21.8%). The naive ABN performed the worst in terms of recovering edges, achieving a proportion of 17%. These results illustrate how a mixed-effects parameterization is better suited to model the within-individual clustering inherent to observational N-of-1 data. The mixed-effects ABN and stepwise models showed similar variation with a standard deviation of 0.16 while the naive ABN had a slightly lower variance of 0.1.

**Table 2:** Aggregated performance measures by structure learning method.

	Matched	SHD	Extra	Missing	Flipped
Naive ABN	0.17 (0.1)	2.125 (0.91)	1.141 (0.88)	0.83 (0.1)	0.155 (0.1)
Mixed-effect ABN	**0.324 (0.16)**	**1.898 (0.83)**	**1.139 (0.85)**	**0.676 (0.16)**	**0.084 (0.07)**
Backward stepwise	0.218 (0.15)	2.3 (0.98)	1.332 (0.97)	0.782 (0.15)	0.186 (0.13)
Forward stepwise	0.282 (0.16)	2.612 (1.26)	1.685 (1.27)	0.718 (0.16)	0.209 (0.14)

Results are aggregated from all 108 parameter permutations, where 5 independent networks were generated for each permutation. Bold numbers indicate best performance in a given metric. All numbers are formatted as “mean (standard deviation)” and were rounded to 3 significant digits.

In terms of the normalized SHD, the mixed-effects ABN performed the best, achieving an average of 1.898. The next best models were the naive ABN (2.125) and the backward stepwise model (2.3). It is notable that the variation in the normalized SHD trends upward, as we go from the best to worst models. Table 2 decomposes the normalized SHD into its constituent sub-metrics: extra, missing, and flipped edges. These results convey a nuanced view of why the models may be performing better or worse. The mixed-effects and naive ABNs performed similarly in terms of extra edges and achieved a normalized ratio of 1.139 and 1.141, respectively. However, the mixed-effects ABN outperformed the other methods at lower node set sizes and average number of parents. The stepwise models had more normalized extra edges, which reflects how they are not subject to restrictions on the parent-child relationships as are the BNs. In terms of flipped edges, the mixed-effects ABN attained the lowest normalized ratio of 0.084. This is almost half the ratio of the next best model, the naive ABN, which achieved a ratio of 0.155. The stepwise models perform the worst in terms of flipped edges, but these results indicate that a non-trivial proportion of the missing edges are actually identified; only the parent-child orientation was incorrect.

#### Model performance by network parameter

**Fig 1:**
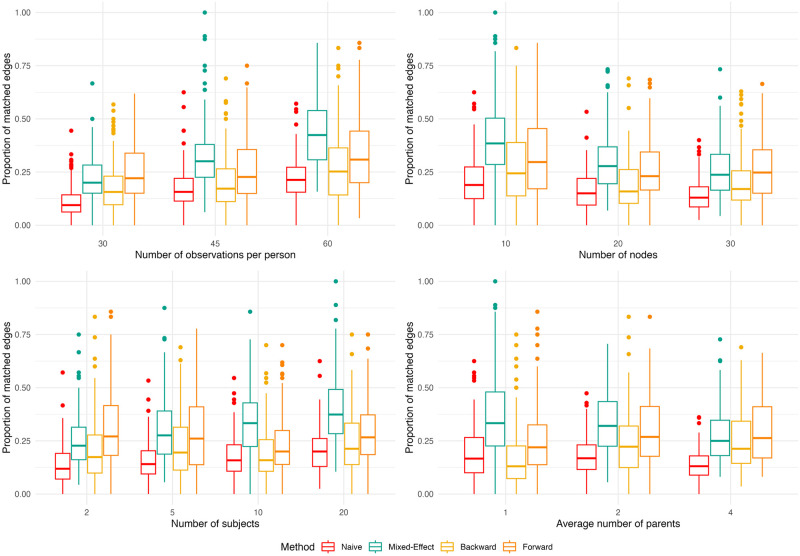
Box plots of the proportion of matched edges for different simulation parameters and structure learning methods.

Fig 1 shows how the proportion of matched edges changed along the network parameters used in the simulation study. All models trended towards a higher proportion of matched edges as the number of observations per individual increases; however, the magnitude of these trends differed. At 30 observations per individual, the two best performing models, the mixed-effects ABN and the forward stepwise model had similar proportions of matched edges; however, at 60 observations per individual, the mixed-effects ABN achieved a median proportion of matched edges of 40% while the forward stepwise model achieved a median proportion of matched edges of 31%. A similar effect is observed as the number of *subjects* is varied. While the mixed-effects ABN median increased from 21% to 37.5% as the number of individuals increased from 2 to 20, no such trend was observed for the forward stepwise model.

In general, model performance on matched edges degraded as the number of nodes increases. The mixed-effects ABN achieved a proportion of 38% with 10 nodes, and this dropped to 25% with 30 nodes. The distribution for matched edges for the naive ABN, and the two stepwise models was identical at 20 and 30 nodes. No meaningful trends were observed in the models as the average number of parents of the true network increased.

**Fig 2:**
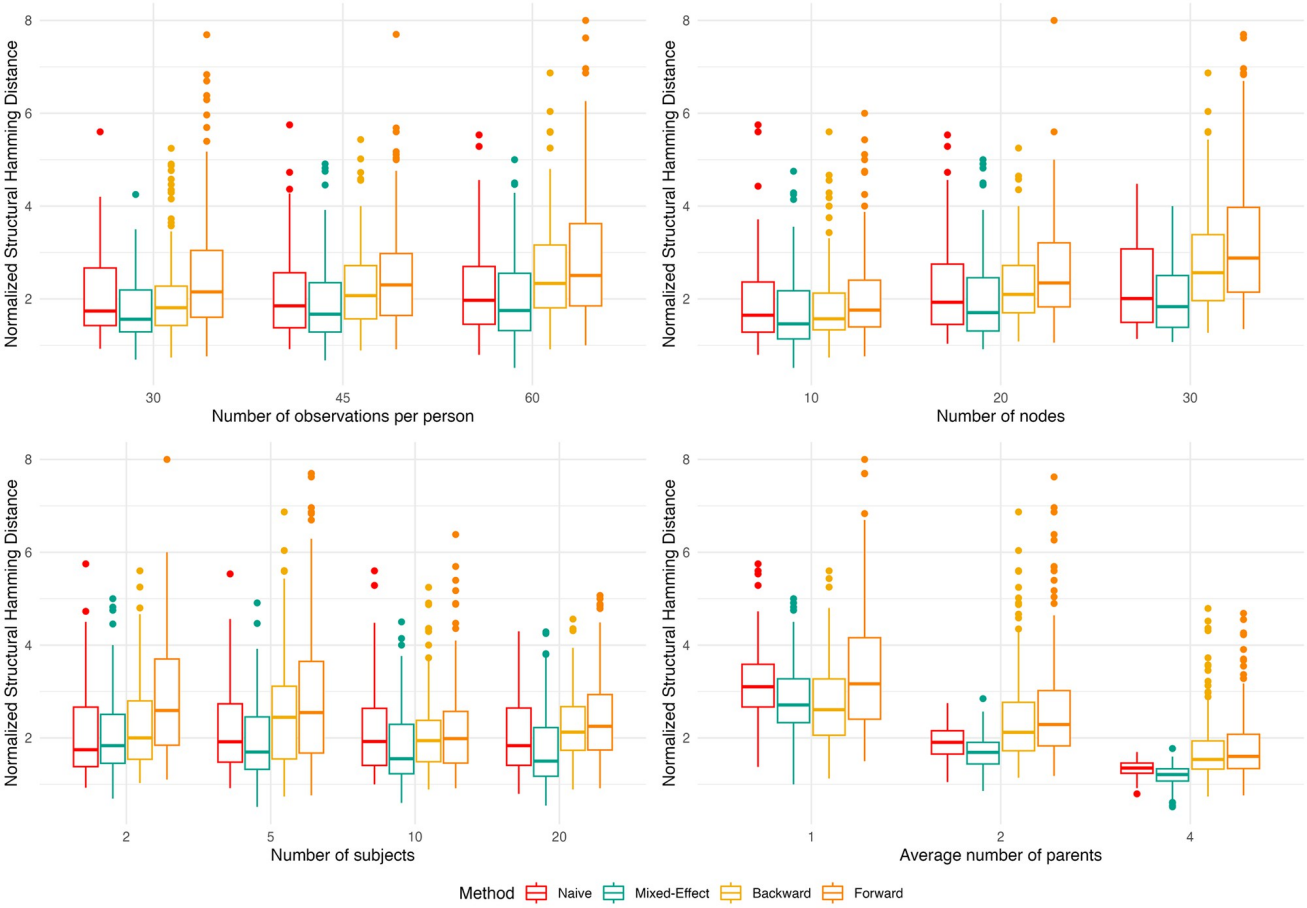
Boxplots of the normalized Structural Hamming Distance for different simulation parameters and structure learning methods.

Fig 2 examines the change in normalized SHD along different network parameters. For the two ABN models, there were no meaningful changes to the median normalized SHD as the number of observations increased; in contrast, the median SHD for the backward and forward stepwise models increased from 1.9 and 2.1 to 2.2 and 2.3, respectively. This suggests that the stepwise models tend to accumulate more mistakes in the network as more data per individual are used. Among the 4 models, the mixed effect ABN benefited from more subjects being used in the analysis. The normalized SHD for the mixed-effect ABN had a decreasing trend as the number of subjects increased, whereas the other models had no such trend. This indicates that the mixed-effect ABN can better leverage the information from more subjects than the stepwise models, despite both using the same model for the local mean. We attributed this discrepancy to the stepwise models’ tendency to pick up extra edges as the amount of data increased. The normalized SHD generally increased as the the number of nodes in the network increases; however, the rate at which this metric increased differs across structure learning method. The ABN models had lower medians, while the stepwise models had noticeably higher normalized SHD. This suggests that the stepwise models also tend to accumulate more mistakes as the network becomes larger. Fig 2 shows that there was a statistically discernible decreasing trend in the normalized SHD as the average number of parents increased. However, we note that this does not imply that structure learning becomes easier as the average number of parents increases. Rather, this was a side effect from normalizing by the number of edges in the true network. The results suggest that the mixed-effects ABN benefit more from denser networks. For instance, the mixed-effect ABN and backward stepwise model performed similarly at an average number parents of 1, but there is a considerable difference in their median normalized SHD when the average number is 4. Overall, the normalized SHD results indicate that our proposed method performs best across reasonable data contexts we might expect for observational N-of-1 designs.

### Empirical study

We considered data from a 12-month randomized controlled trial at Columbia University Irving Medical Center that investigated the bi-directional relationship between physical activity and stress [28]. The study followed 79 healthy adults who reported being intermittent exercisers in the New York City metro area from 2014 to 2015. The first part of the study involved a 6-month observational period to gather from each person on their *personal* predictors for exercise for each individual in the study. This observational data were then used to develop a personalized intervention. Participants were then randomized to receive either general information about exercise or a one-time personalized email based on their results from the observational phase of the study. The study found that individuals receiving a general message had a statistically discernible decrease in the proportion of days they exercised relative to the intervention group. For this paper, only the data from the initial 6-month observational period is used. A more detailed description of the full study can be found in Yoon et. al [28].

Minute-by-minute activity data were measured with a Fitbit Flex, and was categorized by intensity as sedentary, light, moderate, or vigorous. Exercise was defined as any 30-minute period in which at least 80% of the time was spent in moderate or vigorous physical activity (MVPA). A sedentary bout was defined as a bout with 0 counted steps and sedentary intensity.

Stress was measured via three ecological momentary assessments (EMAs) sent throughout the day, and this data were collected via a smartphone-based electronic diary. Each EMA evaluated the degree of stress a person was feeling at that moment as well as the presence of different sources of stress. The sources of stress included arguments, bills, deadlines, tardiness, work, and traffic. We summarized the information from the 3 daily stress assessments into a single daily summary variable for each source as follows. If a person reported feeling any stress from a particular source across all three EMAs, then this was coded as the presence of that source. Otherwise, that stress source was recorded as absent. Missing values for the raw EMA were imputed using linear interpolation of the data from the nearest adjacent observed dates for all individuals. The study data are publicly available on the Open Science Framework.

We fit a mixed-effects ABN on the data similarly to the approach used in the simulation study. That is, each model had a random intercept, and all parent nodes were given a random slope. The BIC was used as the scoring metric in the structure learning phase. We imposed a maximum number of five parents in the resulting network to limit model complexity and reduce computational costs. This number was chosen based on preliminary results in simulation studies; given the potentially large number of model parameters, a maximum of five parents enabled identifiability of the model parameters. The weekend status variable was prohibited from having any parent nodes, and no edges were enforced to be present in the network. Aside from these requirements, the rest of the edges in the network were learned from the data. For our paper, we use a minimal amount of assumptions on the edge set, but one of the benefits of the BN is that prior evidence or expert guidance can easily be incorporated into the network. For other applied contexts, we advise these prior information be utilized to further restrict or add edges to the network *prior* to the data-driven structure learning. After the network is learned, we checked for statistically discernible [29–31] associations at both the population and individual level.

#### Estimated mixed-effects ABN structure and parameters

**Fig 3:**
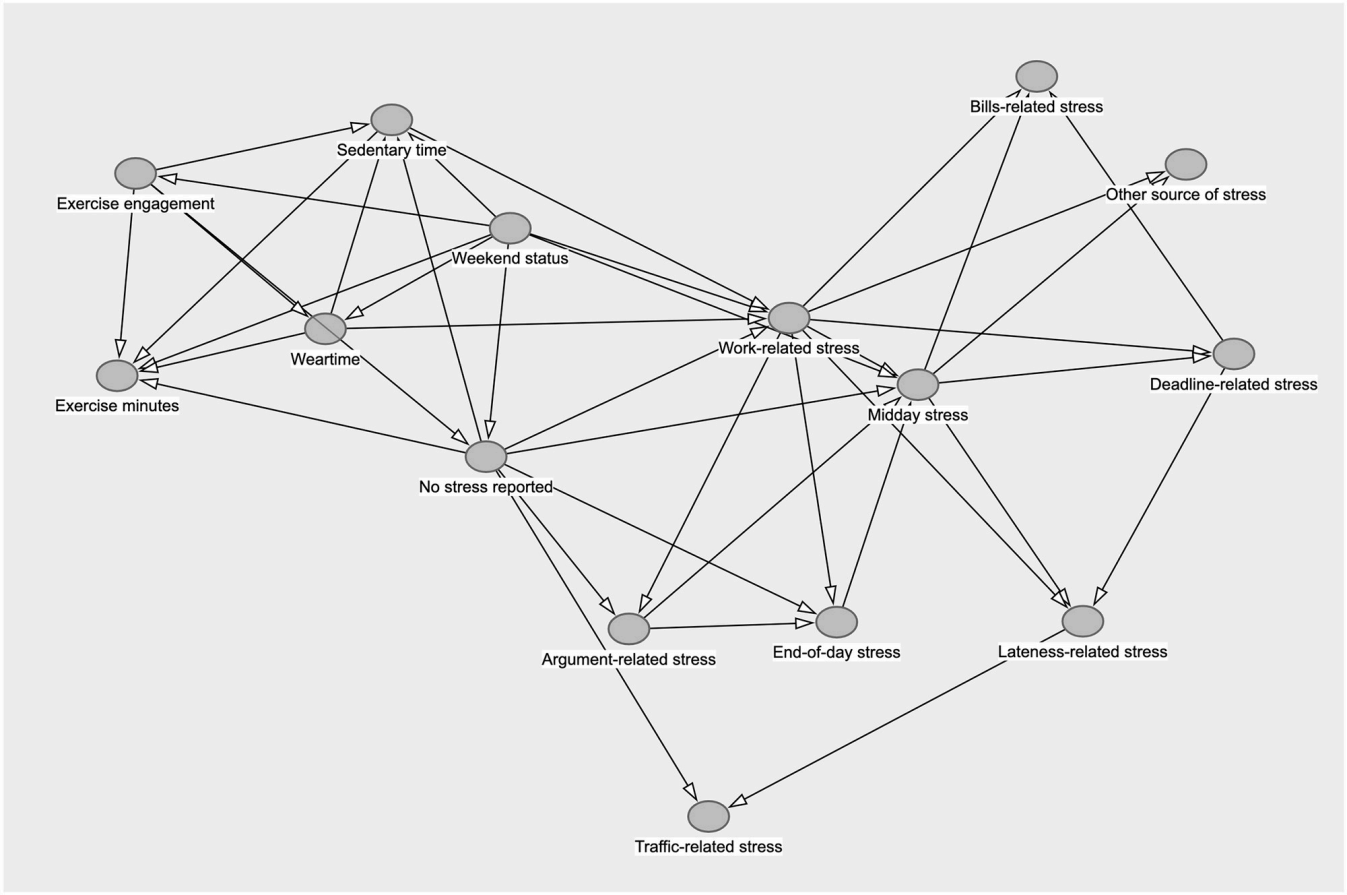
Estimated additive Bayesian network for the empirical study analysis.

Fig 3 shows the estimated, unadjusted ABN for the data. A dictionary explaining all of the variable names can be found in S1 Table in the Supporting Information. Neither exercise engagement nor exercise minutes have specific stress sources as parent nodes. However, the absence of stress was a parent node to exercise time and was associated with a statistically discernible decrease of 2.6 minutes (95% CI: [-4.36, -0.827]). Weekend status was associated with lower odds of exercise engagement, but this effect was not statistically discernible (95% CI: [0.228, 1.13]) On the other hand, the weekend actually increased the amount of exercise for those that partake (7.4 minutes, 95% CI: [4.6, 10.2]). In terms of stress, weekend status was associated with lower odds of reporting work-related stress by 90% (95% CI: [0.07, 0.15]) and average midday stress (-0.17, 95% CI: [-0.25, -0.10]), but was also found to reduce odds of reporting no specific stress source by 43% (95% CI: [0.34, 0.52]).

Based on the simulation study results, we note that some of the learned edges may be spurious or flipped. Therefore, we recommend that the initial learned network be viewed as a start of an iterative process. It is important to remember that the structure learning process is a difficult optimization problem; edges are “learned” because they substantially increase the score (e.g., BIC) for the overall network, but this may not mesh with established literature. Once the network is learned, the edge set should be examined for edges that contradict current scientific evidence or reasonable assumptions about the data. For instance, end-of-day stress was found to be a parent node of midday stress. Since end-of-day stress is recorded later than midday stress, it seems more plausible that this edge should actually be flipped. Furthermore, it is well established that exercise has a protective effect against stress [32–34]. The learned network has the no stress summary variable as a parent to exercise minutes, but it may be of interest to check the associations of exercise as a parent. In this case, a user may opt to restrict any stress-related node from being a parent to certain exercise nodes. Once a network is learned, its structure can be subsequently refined and iterated upon as new literature is established.

Table 3 shows the estimated fixed effects for the local distributions. The learned network suggested that work-related stress plays an important role in an individual’s stress overall. Work-related stress was a parent node to all stress sources except for traffic-related stress, as well as reported midday and end-of-day stress. This relationship trended towards a statistically discernible increase in the odds of reporting specific stress. For example, work-related stress was associated with a 422% (95% CI: [285%, 617%]) increase in the odds of reporting deadline-related stress. The only exception to this was the relationship of work stress to an “other” source of stress, where it was associated with a 66% (95% CI: [68%, 41%]) decrease in the odds. This suggests that future interventions should target work-related stress since the network suggests it can have downstream effects on more specific types of stress.

**Table 3:** Parameter estimates and 95% confidence intervals for all the local distributions in empirical study ABN.

Child	Parent	Regression Coefficient
Exercise engagement	Weekend status	-0.0665 (-0.26, 0.127)
Exercise minutes	Exercise engagement	57 (55.3, 58.8)
	Weekend status	7.45 (4.68, 10.2)
	Weartime	0.0615 (0.0494, 0.0737)
	Sedentary time	-0.103 (-0.123, -0.0832)
	No stress reported	-2.6 (-4.36, -0.827)
Midday stress	No stress reported	1.45 (1.21, 1.7)
	Argument-related stress	0.875 (0.67, 1.08)
	Work-related stress	0.657 (0.519, 0.795)
	End-of-day stress	0.105 (0.0789, 0.131)
	Weekend status	-0.177 (-0.254, -0.101)
No stress reported	Weekend status	-0.577 (-0.737, -0.416)
	Exercise engagement	-0.211 (-0.353, -0.0683)
Sedentary time	Weartime	0.46 (0.431, 0.49)
	Weekend status	-8.9 (-25.7, 7.94)
	Exercise engagement	-57.7 (-73.8, -41.6)
	No stress reported	-1.38 (-6.23, 3.46)
Argument-related stress	No stress reported	0.402 (0.0917, 0.711)
	Work-related stress	0.51 (0.196, 0.825)
Bills-related stress	Midday stress	0.217 (0.092, 0.342)
	Deadline-related stress	1.75 (1.25, 2.26)
	Work-related stress	0.0148 (-0.472, 0.502)
Deadline-related stress	Work-related stress	1.44 (1.05, 1.82)
	Midday stress	0.282 (0.21, 0.353)
Lateness-related stress	Midday stress	0.138 (0.0851, 0.191)
	Deadline-related stress	1.07 (0.729, 1.41)
	Work-related stress	0.146 (-0.0878, 0.38)
Other source of stress	Midday stress	0.292 (0.19, 0.394)
	Work-related stress	-0.822 (-1.13, -0.512)
Traffic-related stress	Lateness-related stress	1.24 (0.863, 1.61)
	No stress reported	0.313 (0.0701, 0.555)
Work-related stress	Weekend status	-2.27 (-2.62, -1.93)
	No stress reported	1.12 (0.893, 1.34)
	Weartime	0.00156 (0.00102, 0.00209)
	Sedentary time	4.27e-05 (-8.59e-04, 9.44e-04)
End-of-day stress	No stress reported	-0.089 (-0.351, 0.173)
	Work-related stress	0.749 (0.603, 0.895)
	Argument-related stress	0.549 (0.286, 0.812)
Weartime	Weekend status	-62.4 (-76.1, -48.6)
	Exercise engagement	26.1 (18.4, 33.7)

All numbers were rounded to 3 significant digits.

#### Examining individual heterogeneity in the ABN

The ABN depicted in Fig 3 can be interpreted as showing the population level relationships that may be present. individual level associations may differ from the population-level associations, so we were interested in examining these discrepancies. To investigate this, we constructed forest plots of the individual level model coefficients, calculated as the sum of the fixed effect and each individual’s random effects. We constructed 95% bootstrap intervals for the forest plots by bootstrapping 1000 data sets, stratified by individual.

**Fig 4:**
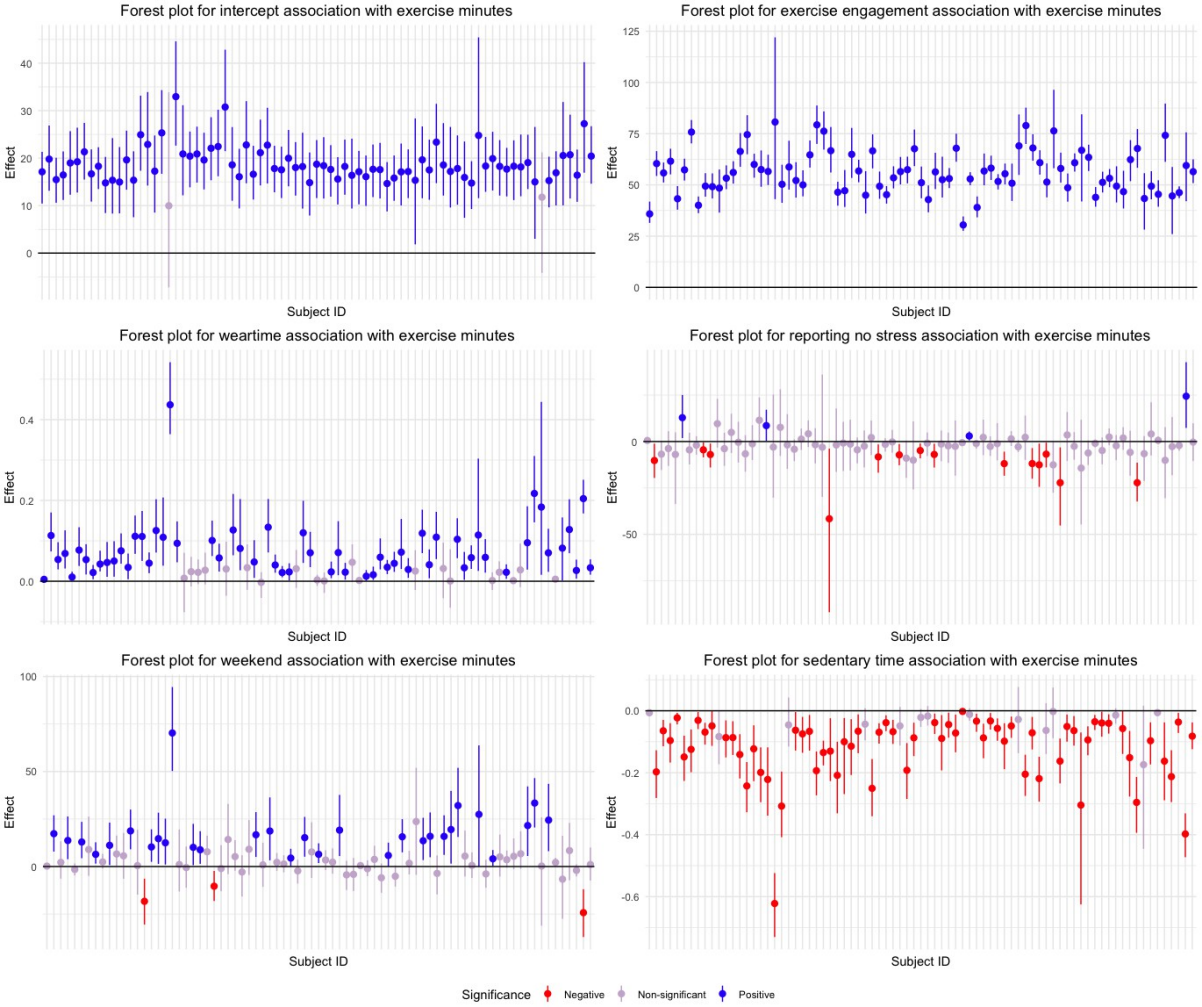
Forest plot of bootstrap 95% intervals for the intercept and all parent relationships to exercise minutes.

To narrow the scope of the analysis, we focus on the local mean of exercise minutes. Fig 4 shows the forest plot for all the parent nodes to exercise minutes and shows the median estimate along with the 2.5th and 97.5th bootstrap quantiles. The intervals are colored to indicate the direction and statistical discernibility of the association. Positive, discernible associations are blue, negative associations are red, and intervals containing zero are purple.

The plots indicate that individuals exercise for different lengths of time, given that they report exercising that day. For example, Subject 1 exercises an additional 35 minutes during an exercise bout, but Subject 7 exercises for more than 75 extra minutes. The forest plot for reporting no stress indicates that reporting no stress does not have an association with more exercise, but four people have a statistically discernible positive association while fourteen have discernible negative effects. A similar result is observed in the weekend effect; most people do more exercise on the weekend, but there are three individuals who don’t. These plots highlight the importance of taking individual level associations into consideration. The estimated fixed effect in the ABN suggests an overall positive association, but some individuals do not follow this trend. We also examine the forest plots for weartime, where the individual associations tend to favor one direction, but still display considerable heterogeneity. Exercise engagement tends to be positively associated with weartime, whereas the weekend has a negative association. In both cases, some people have a statistically discernible association whereas others do not. These results demonstrate that it is possible for the population to have a net direction in the association overall, but for the individuals to differ in its magnitude. These forest plots may be found in S1 Fig in the Supporting Information. These observational findings demonstrate the usefulness of personalizing future interventions to individuals using N-of-1 approaches.

## Conclusion

In this article, we propose a mixed-effects additive Bayesian network as a novel model selection method for data from an observational N-of-1 design. Given the ubiquitous nature of sensors on smartphones and wearable devices, observational N-of-1 designs will become more widespread [35]. This motivates expanding the analytic toolkit that researchers can use.

We recommend ABNs, which can model both population level and individual level associations, which is useful if we pool the data from multiple N-of-1 studies [7]. This method shows promise for identifying determinants of behavior and can guide future development of data-driven interventions. Based on simulation studies, we have shown that mixed-effects ABNs perform the best at recovering network structure relationships compared to other common models. However, this task remains a difficult one, even with large sample sizes.

We applied this approach to a published observational N-of-1 study and discovered how different stress and activity variables were interrelated. We found that work-related stress was associated with other forms of stress, signifying that it may be an impactful target for future research on stress and exercise. Weekend status was found to be associated with all physical activity variables, but its association varies across individuals. By examining the individual variation around the local means, we can ascertain which predictors have considerable levels of heterogeneity that should be taken into account in future studies.

Our method offers a new approach for modeling complex relationships in a multivariate system when we expect these relationships to vary between study units (e.g., subjects). However, this method is computationally demanding, especially with a large node set. For instance, a mixed-effects Bayesian network with 30 nodes takes several hours to estimate with a Macbook Pro with a 2.3GHz quad-core Intel Core and 8 GB of RAM. We limited the maximum number of parents to decrease computational demand and enforced sparseness in the resulting networks. The acyclic nature of the ABN prevents cycles from being considered. Thus, if a reader suspects a bi-directional relationship, they may need to enforce restrictions to ensure that only one parent-child orientation can be considered. We recommend that interested researchers use a small to moderate number of variables in their own analyses. Bootstrapping is recommended to prune the learned edge set of a Bayesian network, but due to computational burden, this was untenable for the empirical study. Thus, we expect spurious edges in the resulting networks, as was demonstrated in our simulation study. Thus, we advise readers to treat the resulting networks as an educated starting point. Prior evidence, scientific plausibility, and other reasonable assumptions may be used to refine a learned network. Another weakness of this approach is that it cannot consider possible cycles (i.e., feedback loops) in the data due to the acyclic nature of the BNs.

In our study, we choose to enforce that each parent node is given a random slope in the ABN to reflect a belief that individuals will vary along each association. However, it’s possible that no such heterogeneity exists in some cases, resulting in overparameterized models. A possible refinement of our approach could be to develop a two-step process that first establishes an initial edge set using simpler models, and then iterate on this initial set using different mixed-effects models. Future work should also focus on reducing computational burden.

## Supporting information

S1 Table Explanation of variable meanings. Names of variables used in empirical study analysis, along with a description of the meaning of each variable.
(PDF)

S1 Fig Forest plots for weartime. Forest plots using weartime as an outcome.
(TIF)
