# Supplementary material for: Multivariate variable selection in N-of-1 observational studies via additive Bayesian networks
Source: PLoS One. 2024 Aug 26;19(8):e0305225. doi: 10.1371/journal.pone.0305225 (PMC11346654; doi:10.1371/journal.pone.0305225)

S1 Table

Table 1: **Names of variables used in case study analysis, along with a description of the meaning of each variable.**

**Variable**	**Description**
Exercise engagement	Indicator that the subject engage in at least one 30-minute bout of exercise that day
Exercise minutes	Number of minutes spend in moderate or vigorous physical activity (MVPA)
Sedentary time	Number of minutes spent sedentary (sitting or laying down)
Weartime	Amount of time that the Fitbit was worn
Weekend	Indicator that the day of the week was Sunday or Saturday
No stress reported	Indicator that the person reported no sources of stress throughout the day
Work-related stress	Indicator that the person reported at least one instance of work-related stress
Argument-related stress	Indicator that the person reported at least one instance of argument-related stress
End-of-day stress	Reported level of stress from the end-of-day EMA
Midday stress	Average recorded stress level from the the 3 EMA in a day
Lateness-related stress	Indicator that the person reported at least one instance of lateness-related stress
Deadline-related stress	Indicator that the person reported at least one instance of deadline-related stress
Other stress source	Indicator that the person reported at least one instance of stress from an unlisted source
Bill-related stress	Indicator that the person reported at least one instance of bill-related stress
Traffic-related stress	Indicator that the person reported at least one instance of traffic-related stress

S1 Figure

Figure 1: Forest plot of bootstrap 95% intervals for the intercept and all parent relationships to weartime

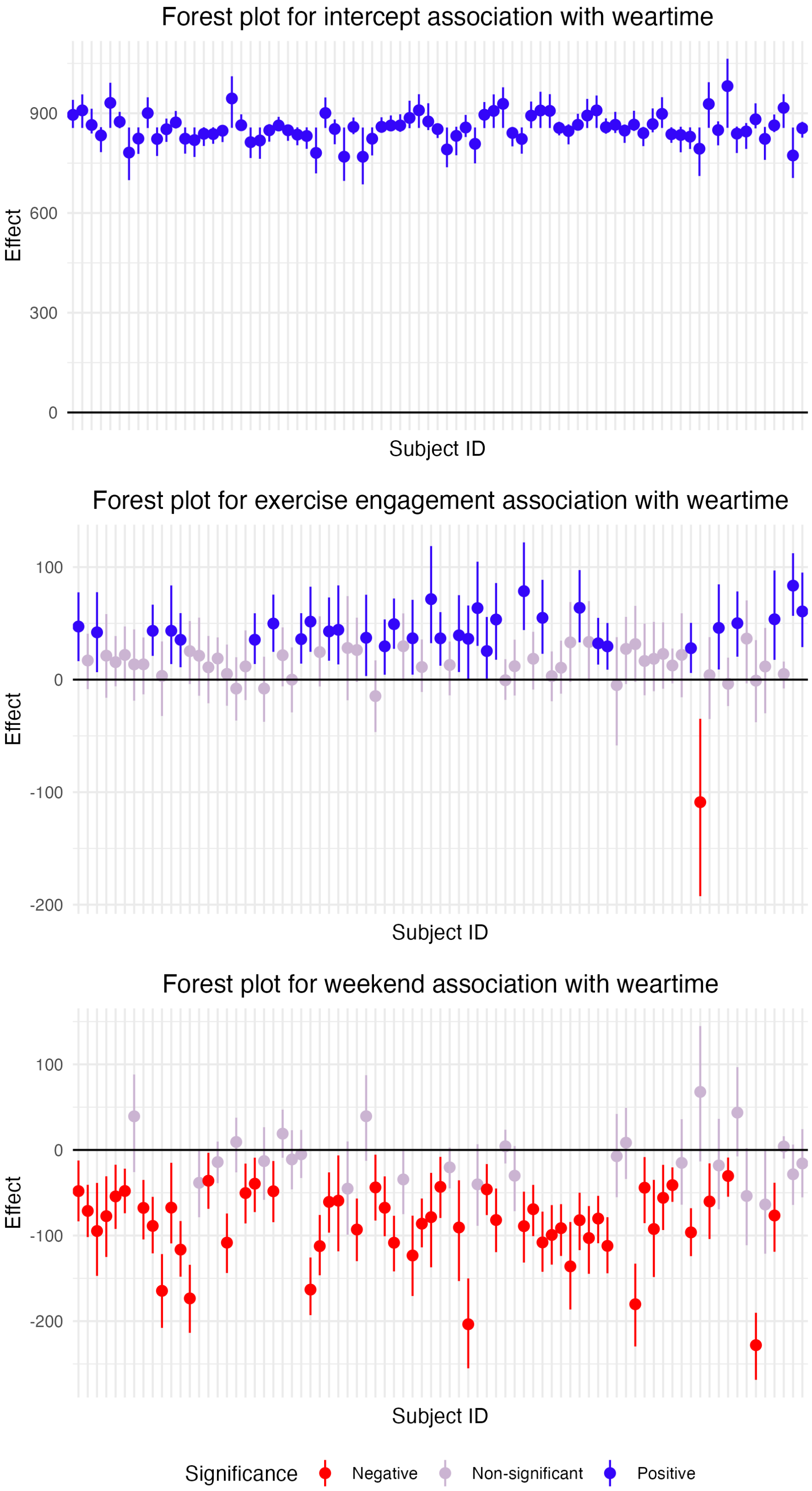

Supplement: S1 Table — Names of variables used in empirical study analysis, along with a description of the meaning of each variable. (PDF) [file pone.0305225.s001.pdf]
