# Supplementary figures and images for: Multivariate variable selection in N-of-1 observational studies via additive Bayesian networks
Source: PLoS One. 2024 Aug 26;19(8):e0305225. doi: 10.1371/journal.pone.0305225 (PMC11346654; doi:10.1371/journal.pone.0305225)

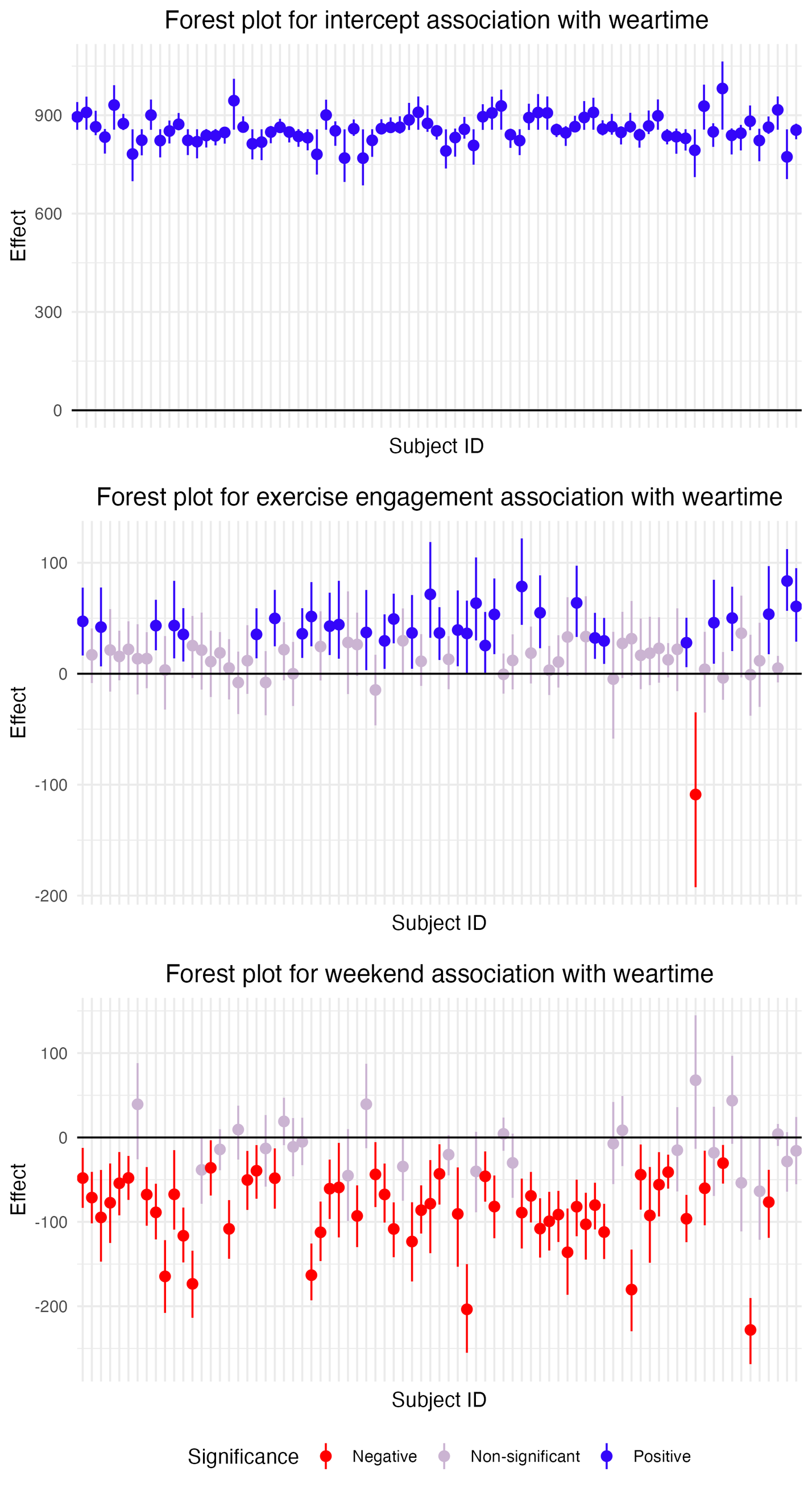

Supplement: S1 Fig — Forest plots using weartime as an outcome. (TIF) [file pone.0305225.s002.tif]
